# Colonic distribution of FMT by different enema procedures compared to colonoscopy – proof of concept study using contrast fluid

**DOI:** 10.1186/s12876-023-02979-x

**Published:** 2023-10-23

**Authors:** Linn Kallbekken Skjevling, Hege Marie Hanssen, Per Christian Valle, Rasmus Goll, Frederik Emil Juul, Øystein Arlov, Peter Holger Johnsen

**Affiliations:** 1 Medical Department, Research Unit, University Hospital of North-Norway Harstad, St. Olavs gt. 70, Harstad, Norway; 2 UIT Arctic University of Norway, Tromsø, Norway; 3 Medical Department, Department of Gastroenterology, University Hospital of North-Norway Harstad, St. Olavs gt. 70, Harstad, Norway; 4 Department of Gastroenterology, University Hospital of North-Norway Tromsø, Sykehusveien 38, Tromsø, 9019 Norway; 5 Clinical Effectiveness Research Group, Oslo University Hospital, Rikshospitalet Gaustad Sykehus (building 20), Sognsvannsveien 21, Oslo, 0372 Norway; 6 Department of Biotechnology and Nanomedicine, SINTEF Industry, Richard Birkelands vei 3 B, Trondheim, 7034 Norway

**Keywords:** Gastroenterology, Fecal microbiota transplantation, Enema, Colonoscopy, Colonic distribution

## Abstract

**Background:**

Fecal microbiota transplantation (FMT) has become an important treatment method in recurrent *Clostridioides difficile* infections and is under investigation as a treatment for several other diseases. FMT’s mechanism of action is assumed to be through alterations of the colon microbiota. FMT can be delivered by several methods, but few studies have directly compared how FMT is distributed in the colon by different methods. Specifically, the proximal distribution of FMT delivered by enema is unknown.

**Methods:**

In eight participants, we administered contrast fluid (CF) with viscosity similar to an FMT in a crossover study design. First, CF was administered by colonoscopy, followed by an abdominal X-ray to visualize the CF distribution. Next, after four to eight weeks, participants were given CF, but as an enema, followed by a positioning procedure. X-rays were obtained before (enema ÷) and after (enema +) the positioning procedure.

**Conclusion:**

Proportion of participants with CF in cecum were 100% after colonoscopy, 50% after enema + and 38% after enema ÷. In the transverse colon, proportions were 100% (colonoscopy), 88% (enema +) and 63% (enema ÷). There were no adverse events.

**Interpretation:**

This study shows proof of concept for the distribution of FMT to proximal colon when delivered by enema. A positioning procedure after the enema slightly improves the proximal distribution. However, colonoscopy is the only method that ensures delivery to the cecum. Studies are needed to see if FMT colon distribution correlates with treatment effectiveness.

**Trial registration:**

The study was retrospectively registered at ClinicalTrials.gov (NCT05121285) (16/11/2021).

**Supplementary Information:**

The online version contains supplementary material available at 10.1186/s12876-023-02979-x.

## Introduction

Fecal microbiota transplantation (FMT) involves transfer of feces from a healthy donor to the gastrointestinal tract of a recipient and has proven to be very effective in treatment of recurrent *Clostridoides difficile* infections. It is believed that FMT work by restoring a healthy colon microbiota in the recipient. Currently, many clinical trials are investigating FMT’s therapeutic potential in other disorders that may be gut microbiota related, such as irritable bowel syndrome (IBS) [1], severe obesity, fatigue related diseases and serious antibiotics-related diarrhea [2].

FMT administration methods vary, and none have consistently shown to be superior to the others [3]. Methods can broadly be categorized as upper (oral) or lower (anal) administration routes. Nasogastric- or nasojejunal tubes, gastroscopy and oral capsules are the most used methods for upper administration, while colonoscopy and enema are the main methods for lower administration. Traditionally, colonoscopy has been the method of choice because it allows simultaneous differential diagnostics (e.g. malignancy or other causes of the symptoms) and because FMT delivery in the cecum is believed to ensure colonization in all colon segments.

FMT by colonoscopy require highly specialized personnel and facilities and is an invasive procedure that can be unpleasant for the recipient. Enema, on the other hand, can be given at the patient´s bedside, is less invasive and put less strain on hospital resources. It is believed that FMT delivered by enema only reach the rectum and sigmoid colon [4, 5], but so far, no published study has assessed the colonic distribution of FMT delivered by enema.

Our hypotheses are that FMT delivered as an enema with positioning (enema +) is superior to enema without positioning (enema ÷) in reaching the transverse colon and cecum, and that FMT distribution after enema + is similar to the distribution when FMT is delivered by colonoscopy.

## Method

### Trial design

This was an assessor blinded, open labeled, single center crossover trial performed at the University Hospital of North-Norway Harstad, Norway. We used contrast fluid (CF) (Barium sulfate suspension 105% w/v, Liquid Polibar Plus, E-Z-EM Canada Inc) as a surrogate liquid for FMT produced in accordance with current European recommendations [6]. In brief, fifty grams of freshly delivered feces from a healthy donor is mixed with 25 mL 85% glycerol and isotonic saline to a total volume of 200 mL. The liquid mix is homogenized and filtered through a strainer with mesh size 0·5 mm, before transfer to four 50 mL Luerlock syringes. Prepared FMT-treatments are frozen and stored at -80 °C. Prior to delivery, frozen FMT-treatments are slowly thawed in a water bath (+ 37 °C) for 60 min before being mixed with 240 mL isotonic saline in an enema bag and transported to the patient for administration [1].

**Fig. 1:**
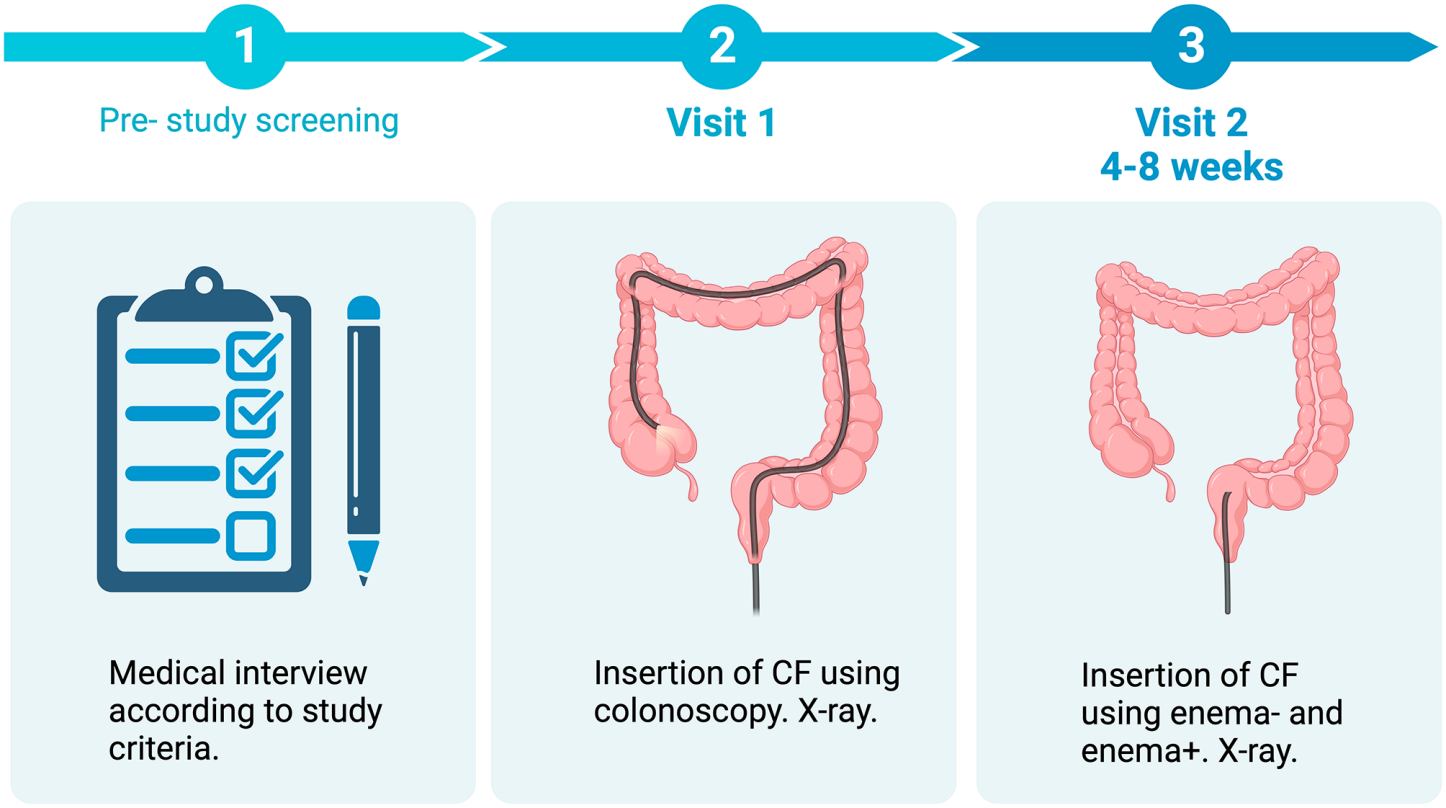
Timeline and characteristics of each visit, created with BioRender.com

The viscosity of the CF was compared to the viscosity of an FMT-treatment (appendix 1). Similar to the procedure for FMT-delivery at the hospital, CF (150 mL) was diluted in 300 mL water before administration, giving a total volume of 450 mL. Timeline and characteristics of each visit is shown in Fig. 1.

**Fig. 2:**
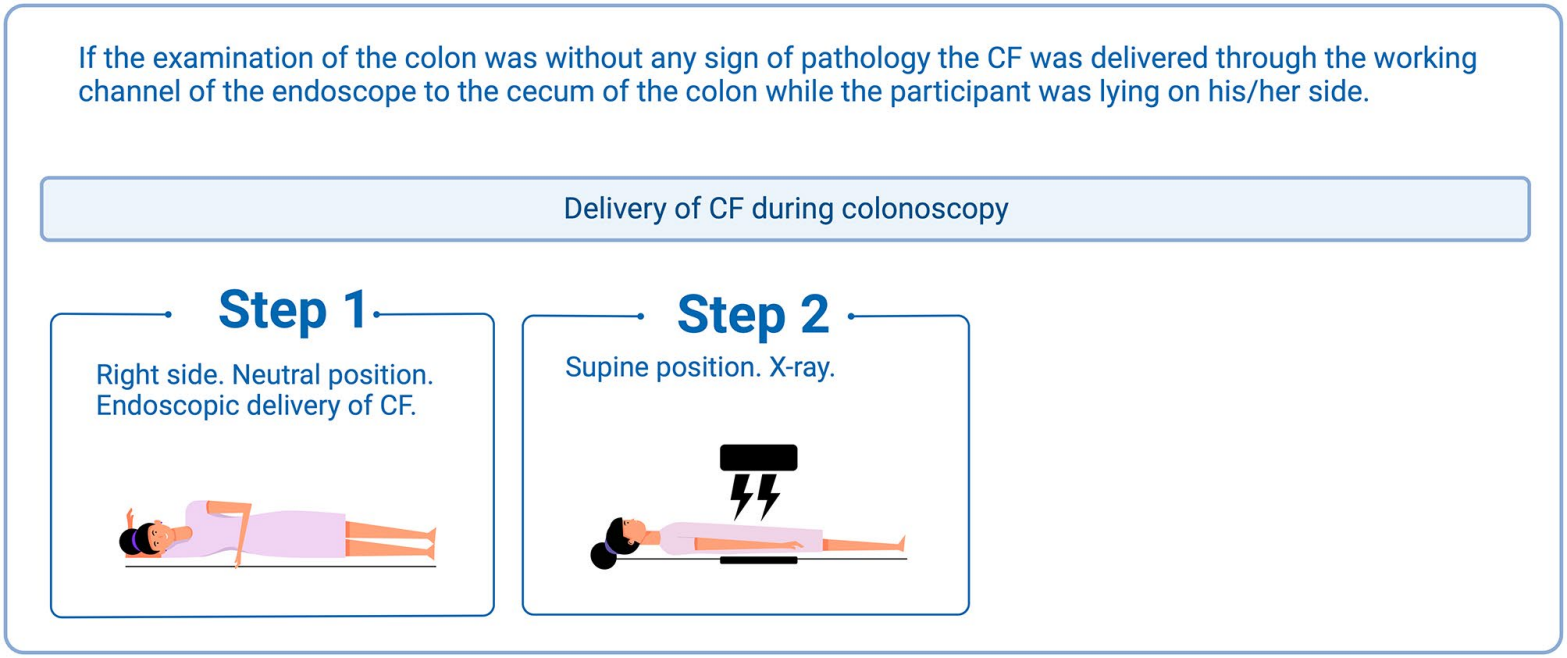
Contrast fluid delivered by colonoscopy, created with BioRender.com

On the first visit, the examiner finished a complete examination of the colon before delivering CF in cecum, followed by an abdominal X-ray (Fig. 2).

**Fig. 3:**
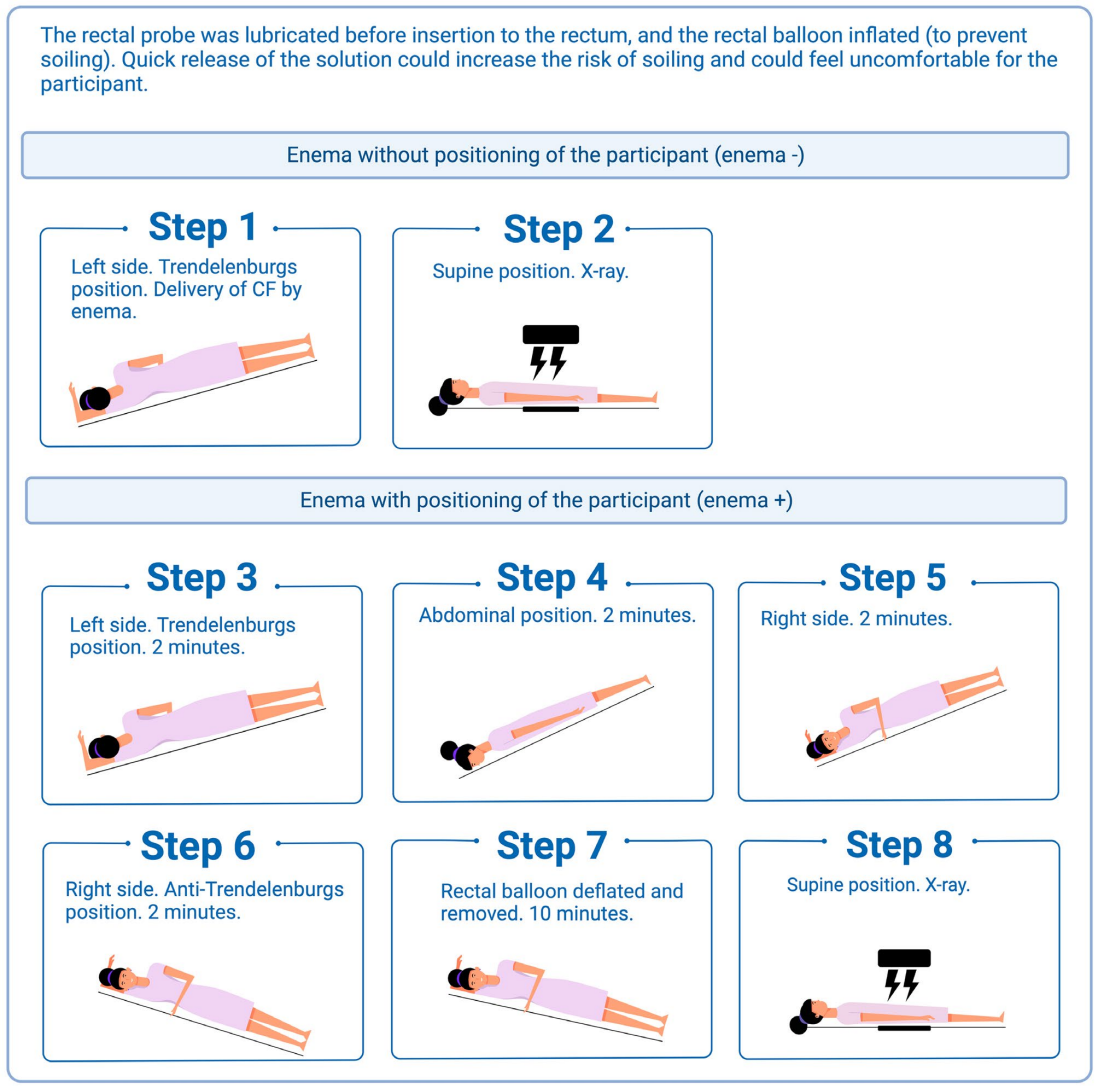
Contrast fluid delivery by enema before and after the positioning procedure, created with BioRender.com

After a washout period of four to eight weeks, participants underwent a bowel lavage with Sodiumpicosulphate/Magnesiumcitrate (Picoprep/Ferring) before returning to the hospital to receive CF, now delivered as an enema (Fig. 3).

Immediately after enema delivery, participants were (with minimal movement) lifted to another table for an X-ray (enema ÷). Next, lying on the bench, participants were instructed to follow the positioning procedure described in Fig. 3, before a new X-ray was performed  (enema +).

### Participants

Patients 18 to 70 years old (median 67) scheduled for a colonoscopy at the gastroenterology outpatient clinic were screened for eligibility. Patients were excluded if they had malignancy, inflammatory bowel disease or obstructive gastrointestinal disease suspected in advance or confirmed during colonoscopy. Other exclusion criteria were use of anticoagulants, symptomatic cardiovascular or lung disease, kidney failure, contraindications to the trial positioning procedure or contrast fluid (Barium sulfate suspension 105% w/v, Liquid Polibar Plus, E-Z-EM Canada Inc), asthma, breastfeeding or ongoing or planned pregnancy. All participants signed informed consent before trial inclusion.

### Outcome and analysis

Our primary outcome was the proportion of participants with CF in the cecum after each administration method. Secondary outcome was the proportion of participants with CF in the transverse colon after each administration method. Proportions were compared using a Fischer Exact test with a significance level of 0·05. We used SPSS [28·0·1·0 (142)] to perform the calculations.

All X-rays were evaluated by two independent radiologists blinded to administration method or procedure (assessor blinding). When the radiologists’ reports differed, a third (blinded) radiologist re-evaluated the X-ray images.

### Ethics

CF was delivered during scheduled colonoscopies necessary for medical purposes. The enema procedure is less invasive compared to a colonoscopy and therefore unlikely to cause any harm in healthy individuals such as our trial participants. Study personnel were trained and equipped to handle any case of anaphylactic reactions to the CF. Participants were exposed to a low radiation dose: one abdominal X-ray is equal to 10 days of background radiation. The trial is registered at ClinicalTrials.gov (NCT05121285).

## Results

A total of 31 participants were assessed for eligibility and ten were found to be eligible. Main medical reasons for exclusion were suspected malignancy (n = four), asthma (n = four), and history of trouble with bowel preparation in earlier colonoscopies (n = two). Among the ten eligible participants, two declined to participate, while the remaining eight participants were included and completed the trial, between 26. October 2021 and 4. April 2022. The study population consisted of three men and five women with median age 67 years. Dilution of CF at 1:2 in water resulted in similar shear viscosity to FMT.

**Fig. 4:**
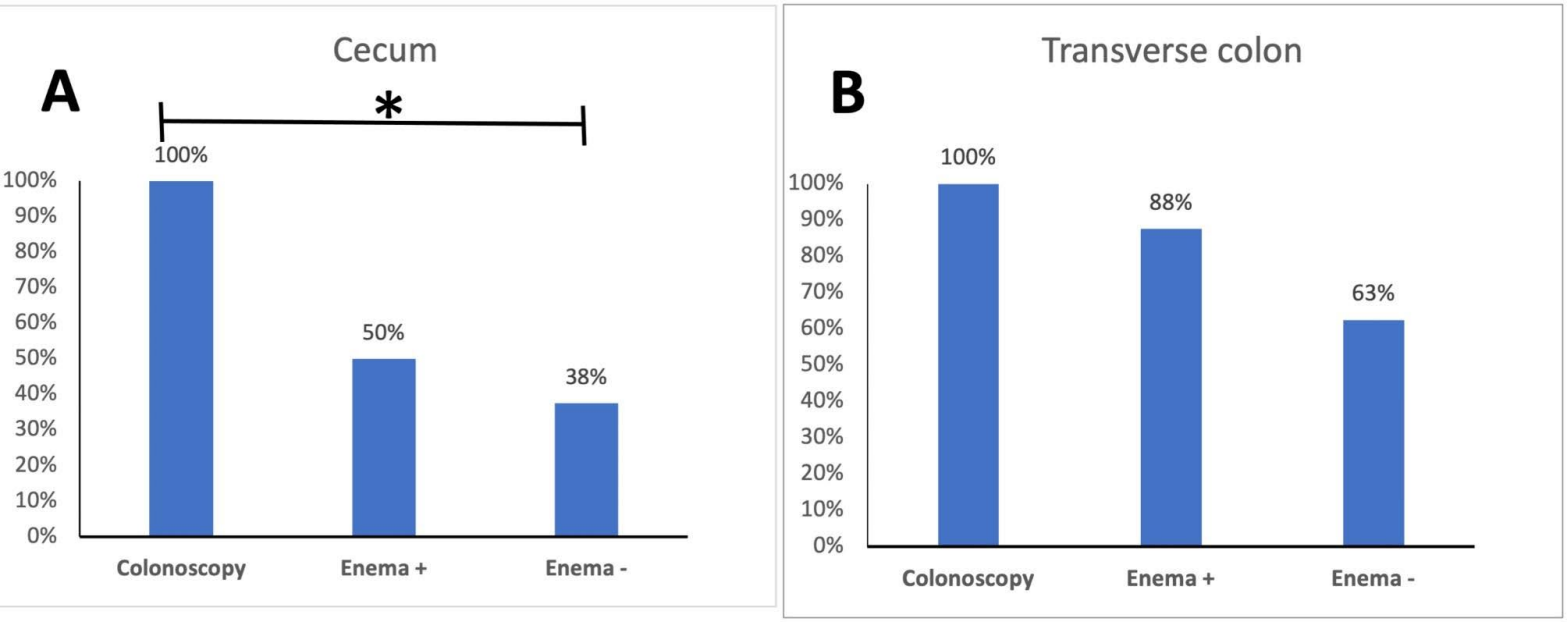
Percentage of participants with visualization of CF by different delivery methods. A: CF visualization in the cecum. B: CF visualization in the transverse colon. * p < 0·05

**Fig. 5:**
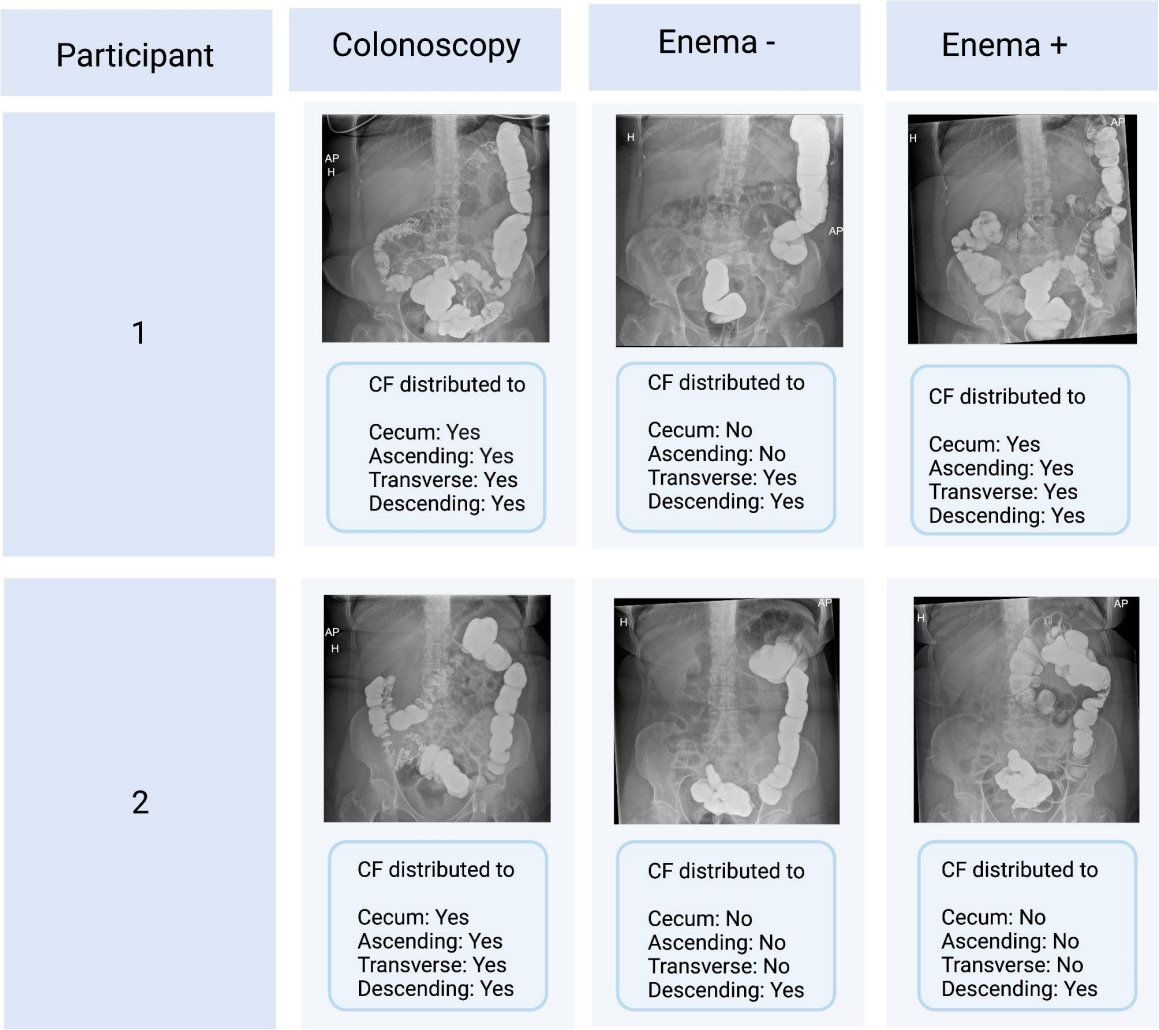
X-ray pictures from two study participants after colonoscopy, enema (-) and enema (+), created with BioRender.com

Proportion of participants with CF visualization in the cecum were 100% after colonoscopy, 50% after enema + and 38% after enema ÷ (Fig. 4a). Colonoscopy compared to enema ÷, but not enema +, had a statistically significant difference in proportions (P = 0·026). Proportion of participants with CF visualization in the transverse colon were 100% after colonoscopy, 88% after enema + and 63% after enema ÷ (Fig. 4b). CF distribution on abdominal X-rays in two study participants are shown in Fig. 5 and all the other X-ray results in appendix 2–4.

All participants tolerated the enema procedure well and there were no adverse events. One of the participants experienced some soiling of CF after the enema insertion, before the first X-ray was performed. During digital exam before enema insertion the sphincter tone was identified as weak. CF was still visualized in the patient’s cecum in both X-ray images.

## Discussion

To our knowledge, this is the first trial to evaluate how fluid similar to FMT is distributed in the colon immediately after delivery by colonoscopy or enema. CF delivered to the cecum by colonoscopy is indeed distributed across colon, which we saw in all participants. Following CF by enema, most patients had CF distributed to the transverse colon, and some even to the cecum, which contradicts the assumption that FMT delivered as enema is only distributed to the distal colon (i.e. rectum and sigmoid colon) [4, 5]. Our results also indicate a slightly improved proximal distribution of FMT by including a standardized positioning procedure.

It is reasonable to believe that better FMT distribution increases the chance of donor microbiota engraftment, which has been shown to be associated with treatment outcome [7]. In this sense, colonoscopy seems to be the best option. If enema is to be used, our results suggest that the enema should be followed by a positioning procedure to improve treatment effect. As enema administration of FMT is less standardized, resulting in differences in distribution depending on which procedure being applied, it may contribute to the difference in treatment effect in studies using enema [8].

Treatment cost, procedure invasiveness and risk of adverse events should be considered when choosing administration method. FMT by enema have the advantage that it can be given bedside, requires little training of the healthcare personnel performing the procedure and involves fewer adverse events than colonoscopy [9]. By being less invasive enema is also likely to cause less patient discomfort. A disadvantage of FMT by enema, compared to colonoscopy, is that it is not possible to know whether the FMT treatment has reached cecum. However, our results show that the FMT is distributed at least to the transverse colon in most patients.

The distribution of FMT delivered by enema may vary because of individual differences in colon anatomy. Studies have shown that the transverse colon is the major determinant of colonic length and that longer transverse colon is more tortuous with sharp-angles [10, 11] which may limit FMT distribution to the proximal colon even with the positioning procedure. Further, the transverse colon is significantly longer in women, older- and thinner adults [11]. Although far too small sample size to conclude, it is interesting to note that the current trial mainly included women (5/8 participants) of older age (median age 67) who were lean. Another factor that may limit FMT distribution is abdominal surgery that leads to adhesions and scar tissue causing anomalies in how the large intestine is positioned. Unfortunately, we did not ask any of the participants if they had undergone any bowel surgery.

Strengths of our trial is the use of a fluid proven to have the same viscosity as FMT, direct comparison of different methods in the same individual (i.e. same anatomy), standardized procedure for administration methods and outcome assessor blinding (X-ray evaluation).

The main limitation**s** to our trial are the small sample size and a design that cannot answer if colon distribution of FMT correlates with treatment effect. The study conducted only an immediate observation of the CF distribution, precluding an evaluation of the duration and quantity of fluid retained in the intestine. Fluid retention time may influence FMT treatment effect, but, to our knowledge, no study has investigated this specifically. An important factor to fluid retention time is probably colonic motility, which changes with older age ^(12, 13)^. We don’t know if there are any differences in how CF, FMT or the different administration methods used in the current study influence motility.

Another limitation is that the enema probe was visualized on the abdominal x-rays in a few patients, which could compromise blinding (i.e. probe was only inserted at enema). As the outcome assessors were not familiar with the enema procedures it is unlikely that this interfered with the blinding. Further, the enema + procedure used in our trial deviates slightly from how we recommend performing the procedure outside the trial: To obtain an abdominal X-ray from both enema ÷ and enema + procedures from one enema delivery, patients were lifted in a supine position after CF delivery by enema and returned to a supine position after the first X-ray (Fig. 3, step 2). Normally we would do the enema + procedure without the extra supine positioning. Lastly, no participant skipped the positioning procedure, which means that the movement of CF in a proximal direction as observed in enema + could simply be an effect of time.

## Conclusion

This study shows proof of concept for the distribution of FMT to proximal colon when delivered by enema. Positioning the patient slightly improves the proximal distribution. However, colonoscopy is the only method that ensures delivery to the cecum. Studies are needed to see if FMT colon distribution correlates with treatment effectiveness.

### Electronic supplementary material

Below is the link to the electronic supplementary material.

Supplementary Material 1

Supplementary Material 2

Supplementary Material 3

Supplementary Material 4

## Data Availability

The data supporting the findings in this research project will be delivered to researchers who provide a sound proposal, on request. The lead authors affirm that the manuscript is an honest, accurate and transparent account of the study being reported. That no important aspects of the study have been omitted. For request of the data please contact Peter Holger Johnsen at peter.holger.johnsen@unn.no.

## List of abbreviations

ØA: Øystein Arlov
CF: Contrast fluid
CFS/ME: Myalgic encephalomyelitis/chronic fatigue syndrome
COLONIZE: Comparative effectiveness of intestinal microbiota versus vancomycin for primary *C. difficile* Infection - randomized Trials
FEJ: Frederik Emil Juul
FMT: Fecal microbiota transplantation
HMH: Hege Marie Hanssen
IBS: Irritable bowel syndrome
LCKS: Linn Christin Kallbekken Skjevling
PCV: Per Christian Valle
PHJ: Peter Holger Johnsen
RG: Rasmus Goll

## Declarations

### Dissemination

Results from this study will be presented at national and international conferences.
