# Supplementary material for: Colonic distribution of FMT by different enema procedures compared to colonoscopy – proof of concept study using contrast fluid
Source: BMC Gastroenterol. 2023 Oct 23;23:363. doi: 10.1186/s12876-023-02979-x (PMC10594821; doi:10.1186/s12876-023-02979-x)
Supplement: Supplementary file 1 — Supplementary Material 1 [file 12876_2023_2979_MOESM1_ESM.pdf]

Appendix 1, Supplementary data: Viscosity measurements of fecal transplant and contrast fluid

Viscosity measurements on the contrast fluid (CF) and fecal transplant were done on a Kinexus rheometer (Netzsch, Germany), equipped with an upper double gap bob geometry (25 mm outer diameter) and a lower double gap system cup. Measurements were carried out over a temperature ramp of 15 to 40 °C, over 5 min, at shear rates of 0.1, 1.0 and 10 s^{-1} and a frequency of 1 Hz. Results are shown below for 1.0 s^{-1}, as no significant differences were observed between the different shear rates for diluted CF and the fecal transplant.

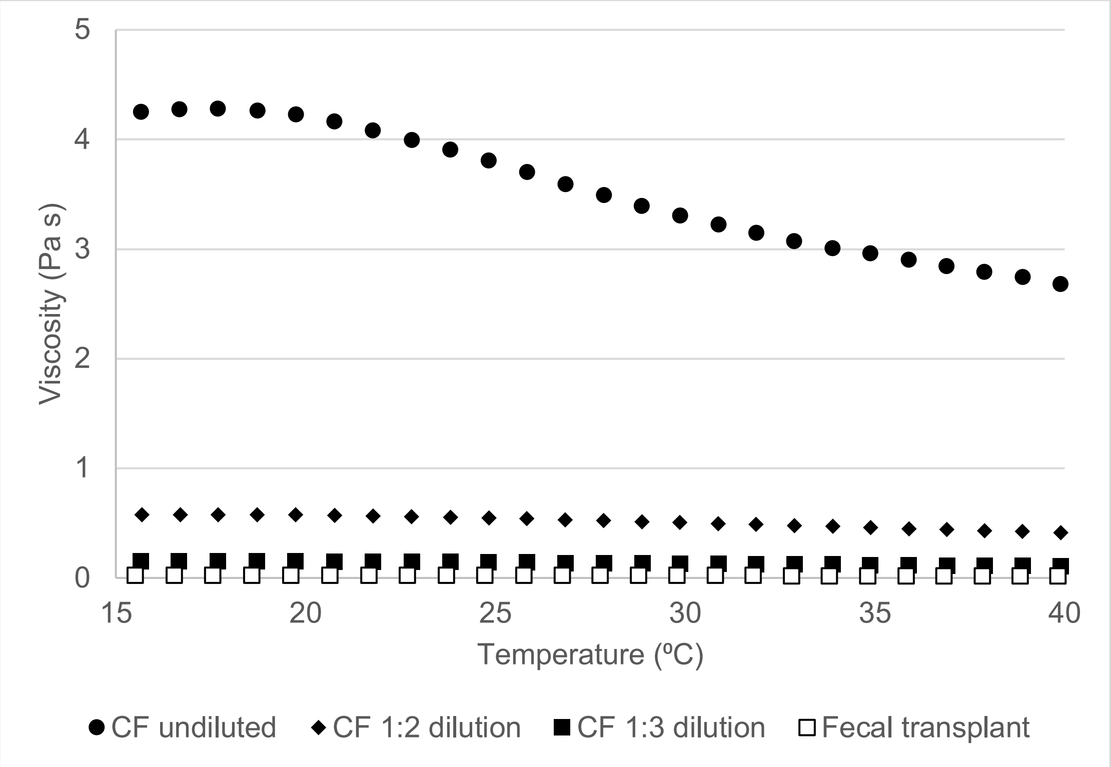

Figure 1: Shear viscosity of undiluted and diluted contrast fluid (CF) and fecal transplant, as a response to increasing temperature. Measurements were carried out at a shear rate of 1.0 s-1 and frequency of 1 Hz.

The results showed that undiluted CF had a high viscosity which decreased slightly with increasing temperature during measurement. This was less prominent for the CF sample diluted 1:2 in water, and not measurable in the 1:3 CF dilution and the fecal transplant. The 1:3 CF dilution showed a slightly higher viscosity than the fecal transplant at an average of 0.1 and 0.02 Pa s, respectively. This difference was, however, not considered to be significant for the clinical study as both samples appeared to have an aqueous consistency upon handling, and as the fecal transplants can show small variations in viscosity depending on the donor and handling of the sample prior to administration.
