# Supplementary figures and images for: Colonic distribution of FMT by different enema procedures compared to colonoscopy – proof of concept study using contrast fluid
Source: BMC Gastroenterol. 2023 Oct 23;23:363. doi: 10.1186/s12876-023-02979-x (PMC10594821; doi:10.1186/s12876-023-02979-x)

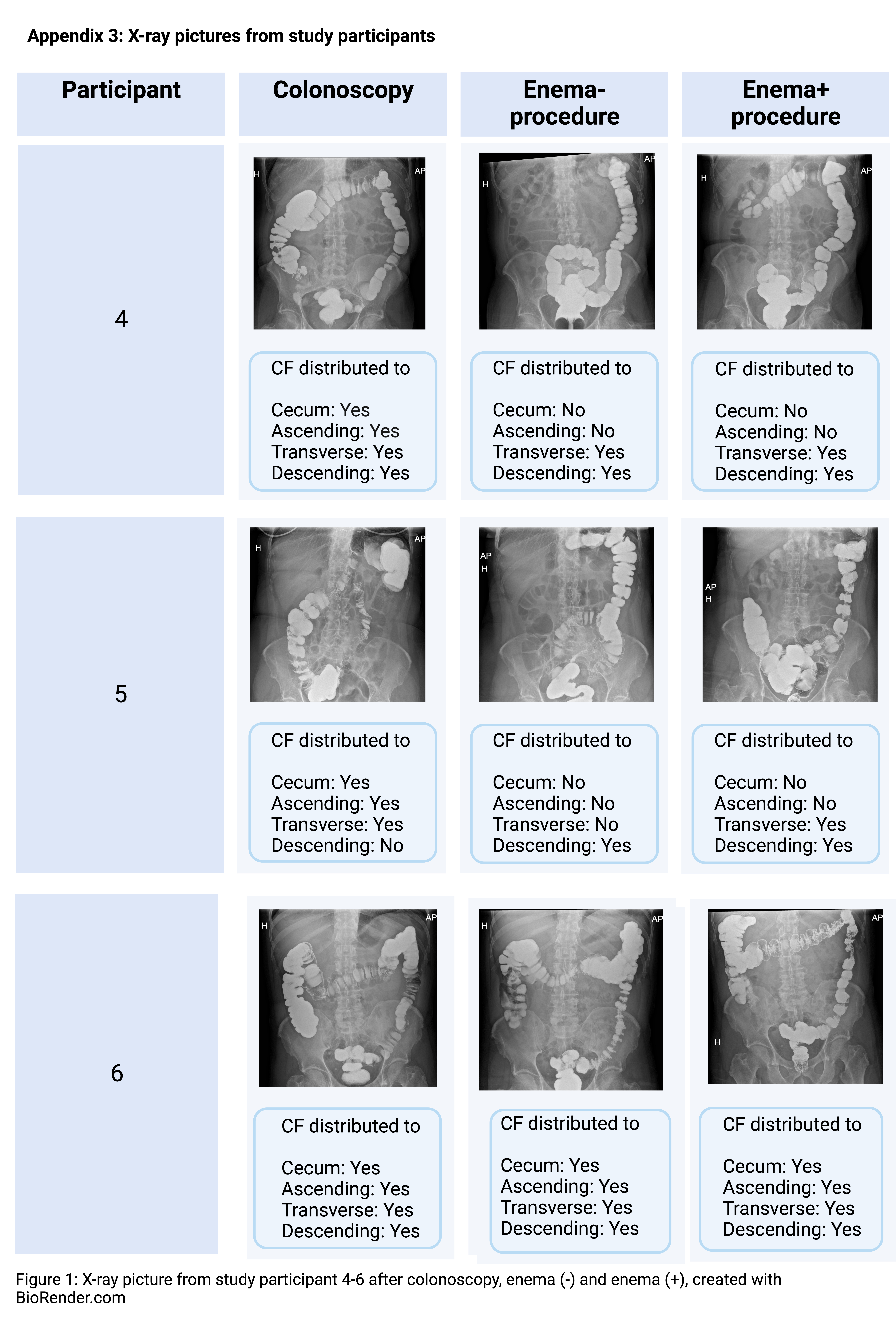

Supplement: Supplementary file 3 — Supplementary Material 3 [file 12876_2023_2979_MOESM3_ESM.jpg]

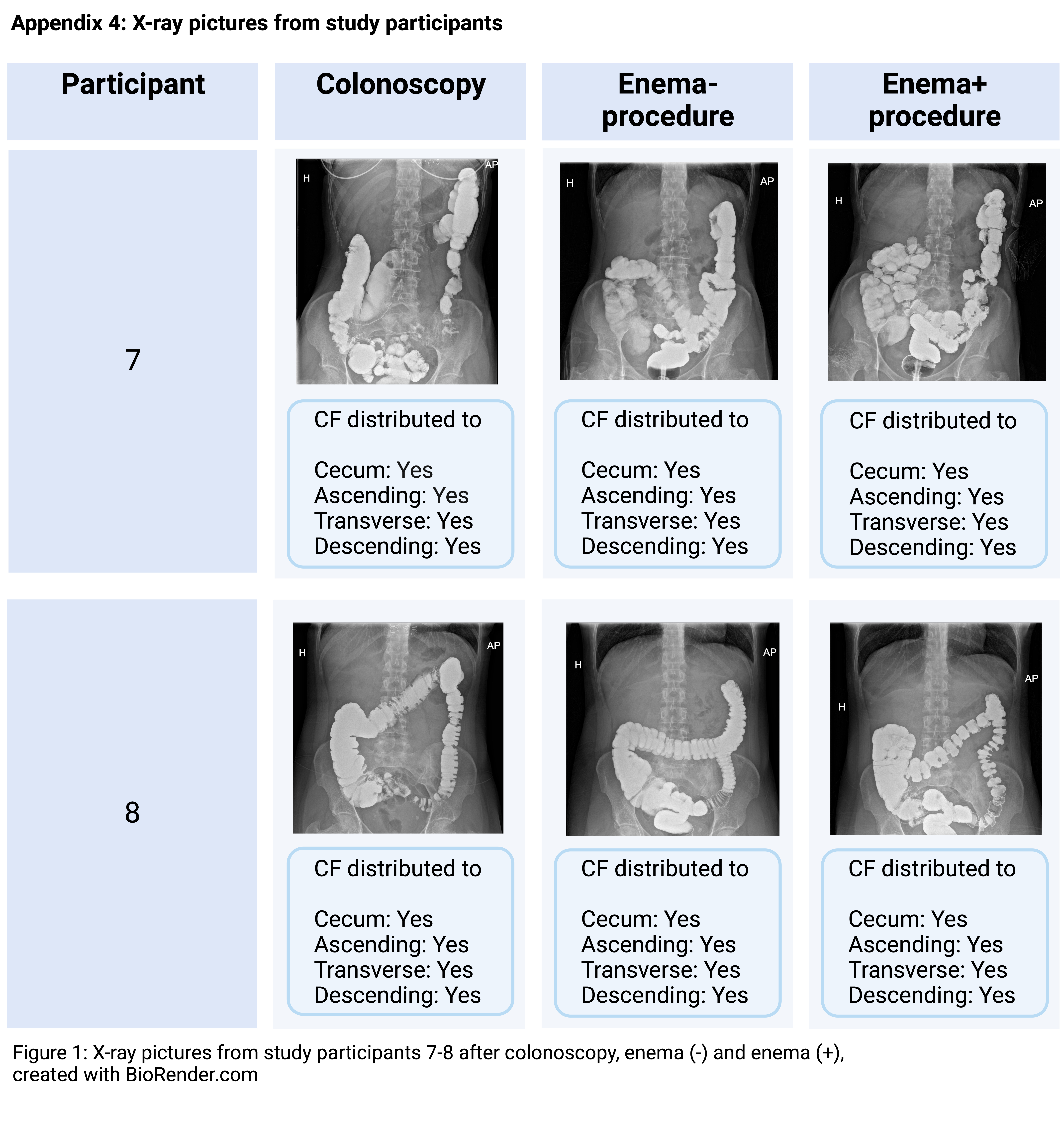

Supplement: Supplementary file 4 — Supplementary Material 4 [file 12876_2023_2979_MOESM4_ESM.jpg]
